# Anti-Oxidant and Anti-Inflammatory Effects of Astaxanthin on Gastrointestinal Diseases

**DOI:** 10.3390/ijms232415471

**Published:** 2022-12-07

**Authors:** Jaeeun Lee, Min-Hyun Kim, Hyeyoung Kim

**Affiliations:** 1 Department of Food and Nutrition, BK21 FOUR, College of Human Ecology, Yonsei University, Seoul 03722, Republic of Korea; 2 College of Health Solutions, Arizona State University, Phoenix, AZ 85004, USA

**Keywords:** astaxanthin, oxidative stress, inflammation, gastrointestinal diseases

## Abstract

A moderate amount of reactive oxygen species (ROS) is produced under normal conditions, where they play an important role in cell signaling and are involved in many aspects of the immune response to pathogens. On the other hand, the excessive production of ROS destructs macromolecules, cell membranes, and DNA, and activates pro-inflammatory signaling pathways, which may lead to various pathologic conditions. Gastrointestinal (GI) mucosa is constantly exposed to ROS due to the presence of bacteria and other infectious pathogens in food, as well as alcohol consumption, smoking, and the use of non-steroidal anti-inflammatory drugs (NSAID). Prolonged excessive oxidative stress and inflammation are two major risk factors for GI disorders such as ulcers and cancers. Bioactive food compounds with potent anti-oxidant and anti-inflammatory activity have been tested in experimental GI disease models to evaluate their therapeutic potential. Astaxanthin (AST) is a fat-soluble xanthophyll carotenoid that is naturally present in algae, yeast, salmon, shrimp, and krill. It has been shown that AST exhibits protective effects against GI diseases via multiple mechanisms. Residing at the surface and inside of cell membranes, AST directly neutralizes ROS and lipid peroxyl radicals, enhances the activity of anti-oxidant enzymes, and suppresses pro-inflammatory transcription factors and cytokines. In addition, AST has been shown to inhibit cancer cell growth and metastasis via modulating cell proliferation-related pathways, apoptosis, and autophagy. Considering the potential benefits of AST in GI diseases, this review paper aims to summarize recent advances in AST research, focusing on its anti-oxidant and anti-inflammatory effects against gastric and intestinal ulcers and cancers.

## 1. Introduction

Reactive oxygen species (ROS) are highly reactive molecules that are produced by the partial reduction of oxygen [1]. Cells endogenously produce ROS as byproducts of normal cellular activity such as mitochondrial oxidative metabolism, xenobiotics metabolism, and bacterial infection [2]. Under physiological conditions, ROS concentrations are tightly regulated. At normal intracellular concentrations, ROS acts as a redox signaling messenger, required for a number of pivotal cellular functions including cell differentiation, proliferation, growth, and apoptosis [3]. Cells are equipped with antioxidative enzymes such as superoxide dismutase (SOD), catalase (CAT), and glutathione peroxidase (GPX), as well as non-enzymatic anti-oxidants such as vitamin C and E, and polyphenols [4]. Once SOD catalyzes the conversion of superoxide anion radical to hydrogen peroxide, CAT or GPX further reduces hydrogen peroxide to water. If these defense mechanisms are disturbed, it causes an imbalance between ROS production and removal, which results in oxidative stress [5]. High levels of oxidative stress can cause the destruction of cellular membranes, lipid oxidation, and DNA damage [3]. In addition, high levels of ROS can initiate the inflammatory process by activating several inflammatory transcription factors such as nuclear factor kappa B (NF-κB), activator protein 1 (AP-1), and signal transducer and activator of transcription 3 (STAT3), as well as cytokines such as interleukin (IL)-1β, IL-6, and tumor necrosis factor-α (TNF-α) [6]. Oxidative stress and inflammation are closely interrelated cellular processes, one of which stimulates the other [7]. Indeed, inflammatory signaling caused by ROS produces a vicious cycle by aggravating ROS production at the site of inflammation [8]. Therefore, oxidative stress and inflammation are two major risk factors for numerous human diseases, and, hence, these are critical therapeutic targets [9].

The stomach and intestine are major digestive organs, where food is chemically and physically processed and nutrients are absorbed [10]. Gastrointestinal (GI) mucosa is constantly exposed to various sources of oxidative stress and inflammation caused by bacterial infection such as *Helicobacter pylori* (*H. pylori*), chronic alcohol consumption, cigarette smoking, and nonsteroidal anti-inflammatory drugs (NSAIDs) [11,12]. Chronic oxidative stress and inflammation mediate the activation of multiple different downstream signaling pathways such as the NF-κB, AP-1 and mammalian target of rapamycin (mTOR), Ras, and Wnt pathways, which are implicated in the development of major GI disorders such as gastric ulcer, ulcerative colitis, gastric cancer, and colorectal cancer [13,14,15,16]. Therefore, there have been extensive efforts to identify new pharmacological agents and bioactive food compounds to effectively reduce oxidative stress and inflammation in the animal models of GI diseases as well as in the clinical setting.

Regarding mucosal protection, adequate organ blood flow is important in maintaining the integrity of GI organs. Appropriate blood flow plays a critical role in protecting and healing the damaged GI mucosa. Previously, Sorbye and Svanes [17] demonstrated that gastric blood flow contributes to protection by supplying the mucosa with oxygen and HCO3^−^, and by removing H^+^ and toxic agents which diffuse from the lumen into the mucosa. Low mucosal blood flow predisposes one to injury, whereas high blood flow protects against injurious agents. The protective and healing-promoting effect of appropriate organ blood flow in the stomach have also been found by other studies [18,19]. In ammonia-induced gastric lesions in rats, the improvement of gastric microcirculation, histamine, and gastric secretion exhibited protection against gastric lesions [18]. In stress ulcer prophylaxis in rats, a gastroprotective effect of histamine accompanied the increase in gastric blood flow [19]. In a trinitrobenzene sulfonic acid (TNBS)-induced colitis model, the administration of obestatin reduced the area of colonic damage, and improved mucosal blood flow in the colon of rats [20]. In acetic acid-induced colitis in rats, Rifaximin and Mutaflor accelerated the healing of colonic damage by reversing the acetic acid-evoked decrease in mucosal blood flow [21]. These studies suggested that improvement in mucosal blood flow in the colon contributes to a decrease in local and systemic inflammatory processes.

Among oxidative stress-mediated GI damage, ischemia/reperfusion (I/R) injury occurs in blood flow-deprived and oxygen-starved organs, followed by the restoration of blood flow and tissue oxygenation [22]. During I/R, xanthine oxidase, NADPH oxidase, mitochondria, and uncoupled nitric oxide synthase produce excessive amounts of ROS. Thus, reperfusion-induced oxidative stress targets for therapeutic intervention against reperfusion-induced organ dysfunction and tissue damage. Although these four enzymes are present in most tissues, xanthine oxidase is enriched in the GI tract. Thus, xanthine oxidase may have a critical role in I/R-induced GI injury [22]. In addition, ferroptosis, a regulated cell death defined by the iron-dependent accumulation of lipid ROS, is closely associated with intestinal I/R injury [23].

For anti-oxidant defense in the GI tract, a gastric hormone, ghrelin, mainly produced in gastric mucosa, reduces the inflammation processes by inhibiting inflammatory cytokine expression and attenuating oxidative stress [24]. In addition, ghrelin exhibits physiological effects, such as the stimulation of growth hormone secretion, gastric secretion, GI motility, and food intake [25]. Due to its anti-oxidant and anti-inflammatory effects, ghrelin shows protective and healing effects in the stomach and other organs. In rat models, ghrelin protected against ethanol-induced gastric ulcers [26,27] and acetic acid-induced gastric ulcers [28]. It protects chemo- or/and radiotherapy-induced oral mucositis [29]. It also attenuates the development of cerulein-induced or I/R-induced acute pancreatitis in rats [30,31]. Exogenous ghrelin promotes the healing of acetic-acid-induced colitis in rats [32,33]. For anti-oxidant activity, ghrelin decreased the level of malondialdehyde and enhanced SOD activity in pancreatic tissue in I/R-induced acute pancreatitis [34], duodenal ulcers in rats [35], dextran sodium sulfate(DSS)-induced colitis [36], and acetic acid-induced colitis [37,38].

Astaxanthin (AST) is a fat-soluble xanthophyll carotenoid that is naturally present in algae, yeast, salmon, shrimp, and krill [39]. Common sources of natural AST include the green algae *Haematococcus pluvialis* and the red yeast *Phaffia rhodozyma* [40]. In the GI, AST has been reported to exert protective effects by reducing bacterial load, modulating immune response, and inhibiting cancer cell proliferation [41,42,43]. These beneficial effects of AST are mostly due to its potent anti-oxidant and anti-inflammatory activity, which is even greater than the same activities in other carotenoids such as lutein and zeaxanthin [44]. Considering AST as a potential nutraceutical in GI diseases, the current paper aims to highlight recent advances in AST research with a focus on its anti-oxidant and anti-inflammatory effects against ulcers and cancers that happen in the stomach and intestine. Here, we will review the pathologic mechanisms of these diseases, and discuss the molecular and cellular mechanisms of AST activity that has been reported in in vitro, in vivo, and clinical trials. The current review was prepared by a comprehensive literature search using multiple databases including Pubmed, Scopus, and Web of Science to identify relevant research.

### 1.1. Anti-Oxidant and Anti-Inflammatory Mechanism of AST

It has been well established that many dietary carotenoids display anti-oxidant capability by directly quenching ROS and indirectly activating the stress response signaling pathways [45]. For example, β-carotene, lutein, and lycopene are major carotenoids that have been extensively studied regarding their anti-oxidant actions of efficiently scavenging singlet molecular oxygen and peroxyl radicals [46]. The potency of carotenoids as anti-oxidants varies depending on their chemical and structural properties as well as how the compounds interact with cell membranes [47]. AST has a unique polar-nonpolar-polar hydrophobic structure that allows the compound to act as a versatile anti-oxidant in cells. The two terminal polar ends contain hydroxyl (OH) and keto (C=O) moieties, which leads to the formation of intermolecular hydrogen bonds between polar groups of phospholipids and the hydroxyl or carbonyl groups. On the other hand, the middle nonpolar region of AST aligns with the inner nonpolar (hydrophobic) parts of the membrane [48]. Therefore, the terminal rings of AST can be located both inside and on the surface of the membrane, and the transmembrane alignment makes AST advantageous to scavenge ROS and lipid peroxyl radicals [49,50,51]. The anti-oxidant potency of AST is even greater than that of other carotenoids [50,52].

There are multiple mechanisms by which AST protects against oxidative stress. Most importantly, AST directly scavenges ROS by donating electrons and by neutralizing free radical into a non-reactive form [41]. Specifically, a series of the double bonds of the carbon scaffold in the non-polar area of AST effectively facilitates a removal of electrons from ROS by transporting the electron to its carbon chain, which breaks free radical chains and prevents lipid peroxidation [11,53]. Due to its transmembrane alignment in the lipid bilayer, AST can synergistically interact with other anti-oxidants such as vitamin C, being an acceptor for the radical cations [54]. AST is also located in the mitochondrial membrane, not only at the cell surface membranes. Therefore, AST modulates the release of cytochrome c and pro-apoptotic factors [41]. In an indirect manner, AST upregulates anti-oxidant signaling pathways and thus controls the mitochondrial redox state and membrane integrity [55]. The ROS-quenching activity of AST also confers protection against inflammation [56]. In various experimental models, AST has been shown to suppress inflammatory transcription factors including NF-κB [57,58], mitogen-activated protein kinases (MAPK) [59,60], nuclear factor erythroid-related factor 2 (Nrf2) [61], and phosphatidylinositol-3-kinase (PI3K) pathways [62]. AST also modulates the expression of pro-inflammatory cytokines and chemokines such as IL-6, IL-1β, TNF-α, vascular endothelial growth factors (VEGFs), and interferon- γ (IFN-γ) [56].

### 1.2. Bioavailability of AST

AST is a fat-soluble compound; therefore, its absorption process is similar to that of lipid absorption. When given orally, xanthophyll carotenoids, including AST, are mixed with bile acid and form micelles in the intestine, which are partially absorbed by intestinal mucosal cells [63]. The micelles, including AST, are then incorporated into chylomicrons by intestinal mucosal cells and enter circulation through the lymphatic system. During circulation, micelles are digested by lipoprotein lipase by which AST can be transported into various peripheral tissues [63,64].

The possible health benefits of AST can be exerted only when the compound has enough bioaccessibility and uptake efficiency by intestinal cells [65]. Recent studies have reported that the uptake efficiency of carotenoid is partly mediated by facilitated diffusion [66,67]. Several lipid transporters of the apical or basolateral sides in the epithelial layer serve as carotenoid transporter proteins, as it is known that carotenoids and lipids exhibit common mechanistic absorption in the intestine [65]. The scavenger receptor class B type I (SR-BI), a cholesterol transporter in enterocyte apical membrane, and cluster determinant 36 (CD36), a fatty acid transporter, have been suggested to be involved in the cellular uptake of some carotenoids such as β-carotene, α-carotene, lutein, and lycopene [68]. However, the roles of these transporters in the uptake and absorption of AST remain to be studied.

The absorbability and tissue distribution of AST have been studied in multiple experimental models. In Balb/c mice, it has been shown that the relative absorption ratios of 13-*cis*, all-*trans*, and 9-*cis* AST isomers were 11.4%, 71.4%, and 17.1%, respectively, in the small intestinal wall; 51.8%, 41.6%, and 6.6% in blood plasma; 62.5%, 30.6%, and 6.9% in the liver [69]. Petri and Lundebye [70] found that the organ distribution of AST in rats after two weeks of oral application was the highest in the spleen, kidneys, and adrenals, whereas the liver concentration was unexpectedly low. Similarly, Singh et al. [71] revealed that AST has the highest accumulation in the spleen, followed by the kidney, heart, and lung, and was the lowest in the liver. This may indicate that, when AST is formulated into emulsion, the compound will have the least activity in the liver compared to other organs. The distribution of AST in plasma showed that approximately 36–64% of AST is present in very low-density lipoproteins (VLDL) and chylomicrons, while low-density lipoprotein (LDL) and high-density lipoprotein (HDL) contained 29% and 24% of total AST, respectively [72]. According to clinical research, the plasma concentration of total AST in three middle-aged male subjects after oral ingestion of a single meal containing 100 mg AST equivalents of AST fatty acyl diesters reached a maximum of 0.28 mg/L at 11.5 h [73].

## 2. Gastric Ulcer

Gastric ulcer is characterized by a deep necrotic lesion in gastroduodenal mucosa with a break in the normal gastric mucosa integrity, which penetrates through the muscularis mucosa into the submucosa [74]. It involves prominent histologic and ultrastructure abnormalities, including reduced height, marked dilation of gastric glands, poor differentiation and/or degenerative changes in glandular cells, increased connective tissue, and disorganized microvascular network [75]. Although the underlying molecular mechanisms need to be elucidated, ROS-mediated gastric inflammation and cell damage have been reported to be highly involved in the development of gastric ulceration. Oxidative stress is a central event in the progression of many inflammatory diseases, including GI inflammation [76]. In the stomach, ROS is produced in response to various triggers such as alcohol consumption, cigarette smoking, ultraviolet radiation, bacterial infection, and the ingestion of NSAIDs [77]. Among them, gastric ulcers are mainly caused by oxidative stress induced by an infection of *H. pylori* or the ingestion of NSAIDs, such as ibuprofen and aspirin in humans. In response to *H. pylori* infection or NSAIDs ingestion, neutrophils are recruited to the inflammatory region, which consequently produces ROS and reactive nitrogen species (RNS). The sources of radicals include mucosal xanthine oxidase and nicotinamide adenine dinucleotide phosphate (NADPH) oxidase which are present in the resident leukocytes and gastric epithelial cells [78]. Increased ROS activate NF-κB and AP-1 which are redox-sensitive transcription factors that upregulate inflammatory genes, and subsequently increase the expression of inflammatory cytokines such as IL-8 and adhesion molecules [79]. Thus, the severity of inflammation within the gastric mucosa is correlated to the oxidative stress.

### 2.1. The Effects of AST on H. pylori–Induced Gastric Ulcer

*H. pylori*, a gram-negative bacterium, is highly associated with the development of GI diseases such as gastritis, peptic ulcer, and gastric carcinoma [80]. According to the global burden of disease estimates, approximately 50% of the world’s population are infected with *H. pylori* [81]. The prevalence of gastric and duodenal ulcers has decreased recently in western countries, since *H. pylori* infection is steadily falling due to improved hygiene and increased antibiotic use [82]. However, still, approximately 15% of the *H. pylori*-infected population develops an ulcer and the risk of disease is influenced by virulence of the *H. pylori* strain, host genetics, and host environment [83]. *H. pylori* can damage the protective lining of the stomach and small intestine, which allows the acid to induce an open sore, leading to gastric ulcer and duodenal ulcer, respectively. Possession of the cytotoxin-associated gene A (CagA) and the production of a vacuolating cytotoxin encoded by the vacuolating cytotoxin A (VacA) are associated with increased risk for atrophic gastritis, gastric ulcer, and gastric cancer pathogenesis [84]. Recent serological studies have shown that the *H. pylori* strain with CagA enhanced gastric epithelial proliferation and apoptosis leads to tyrosine phosphorylation of the CagA protein, inducing an increased inflammatory cell response and high levels of IL-8 [85]. *H. pylori* infection stimulates activation of inflammatory mediators and the release of inflammatory mediators including IL-1β and IL-8 in gastric mucosa of *H. pylori*-infected patients [86,87,88,89,90]. The expression of pro-inflammatory cytokine is involved in a variety of cellular activities, such as inflammatory response and dysregulation of gastric acid secretion [91]. Thus, *H. pylori* infection results in severe gastric mucosal damage.

AST has been demonstrated to protect gastric epithelial cells against *H. pylori* infection by reducing ROS generation and cytokine production. In *H. pylori*-infected gastric epithelial cells, Kim et al. reported that AST treatment (1 or 5 µM; 3 h) activated peroxisome proliferator-activated receptor-γ (PPAR-γ) and induced expression of its downstream anti-oxidant gene such as catalase. Thus, AST suppressed intracellular and mitochondrial ROS production and ROS-mediated NF-κB activation and IL-8 expression [92]. Mechanistically, AST prevented *H. pylori*-induced mitochondrial dysfunction and adenosine triphosphate (ATP) depletion via its anti-oxidant capacity in gastric epithelial cells. The research group also showed that AST (1 or 5 µM; 3 h) prevented *H. pylori*-induced decreases in superoxide dismutase 2 (SOD2) level and SOD activity, and reduced mitochondrial ROS in gastric epithelial cells [93].

There has been several in vivo studies using *H. pylori*-infected mouse models. In C57BL/6 mice inoculated with *H. pylori*, feeding a standard chow diet supplemented with AST (5 mg/kg BW; 7 weeks) significantly attenuated oxidative damage to gastric mucosa cells by reducing the *H. pylori*-induced increase in lipid peroxide (LPO) production, myeloperoxidase (MPO) activity, expression of the inflammatory cytokine IFN-γ, and oncogenes such as c-myc and cyclin D1 [94]. As a result, AST-supplemented mice exhibited lower levels of *H. pylori*-induced neutrophil infiltration, hyperplasia, and histologic changes in gastric mucosa compared to those of non-AST-received mice. Recent studies have reported that AST exerts antimicrobial activity against *H. pylori* infection by shifting the pro-inflammatory T helper type 1 (Th1) response towards an anti-inflammatory Th2 response [95], both of which are predominant subtypes of CD4^+^ T helper cells [96]. The relative balance between Th1 cytokines (e.g., TNF-α, IFN-γ, and IL-12) and Th2 cytokines (e.g., IL-4, IL-5, IL-10, and IL-13) are associated with cancer-related inflammation. The immune response to *H. pylori* infection predominantly activates Th1-cell mediated responses dominated by IFN-γ over IL-4 [97]. In the research conducted by Bennedsen et al., oral supplementation of AST-rich micro algae, *Haematococcus pluvialis*, (200 mg/kg BW; 10 days) in *H. pylori*-infected BALB/c mice markedly reduced bacterial load and gastric inflammation by shifting the T-lymphocyte response from a Th1-dominant to a Th1/Th2-balanced state [42]. This notion was further supported by another study. In *H. pylori*-infected BALB/c female mice, oral administration of AST (10 or 40 mg/kg BW; 6 weeks) elevated the secretion of Th2-type cytokines such as IL-2 and IL-10, leading to a shift towards a balanced Th1/Th2 response in splenocytes [98].

In a randomized clinical trial conducted by Andersen et al., 44 patients infected with *H. pylori* and with functional dyspepsia (average age, 51 yr) received AST capsules (21 patients, 20 mg AST twice daily for eight weeks) or placebo capsules (23 patients, dextrin-filled capsule). The active AST capsule contained algae meal from *Haematococcus puvialis* [99]. Despite no changes in the density of *H. pylori*, AST-treated patients showed a significant up-regulation of T helper cells (CD4), while displaying down-regulation of cytotoxic T cell (CD8), which resulted in a reduction in gastric inflammation. This study was in accordance with the second Helsinki declaration and approved by the local ethics committee. In another randomized double-blind, placebo-controlled clinical study, 42 young healthy females (average age, 21.5 yr) received dietary AST (2 or 8 mg/kg BW for eight weeks). The AST capsule contained oleoresin concentrate from *Haematococcus puvialis* [100]. They showed reduced levels of oxidative DNA damage biomarker (8-hydroxy-2′-deoxyguanosine) and plasma acute phase protein (C-reactive protein) concentration due to the stimulating mitogen-induced lymphoproliferation and increasing natural killer cell cytotoxic activity and total T and B cell subpopulations. All procedures were approved by the Institutional Review Board of Washington State University, USA. Collectively, these results indicate that AST can ameliorate gastric ulcers by modulating immune response and oxidative stress.

### 2.2. The Effects of AST on Other Gastric Ulcer Models

NSAIDs are commonly used analgesic agents due to their anti-inflammatory and pain-relieving properties [101]. However, they can induce some side effects such as ulcerative lesions in the GI tract [102,103]. Naproxen is often used for arthritic patients and utilized as a stimulant to investigate gastric antral ulcer models with erosions and petechial bleeding in the mucosa of the stomach [104]. In rats treated with naproxen, the administration of AST (1, 5, or 25 mg/kg BW; twice daily for three days) showed protective effects against naproxen-induced gastric ulcer by reversing decreased the activities of SOD, CAT, and GPX, and increased the lipid peroxide level to that of untreated normal rats [105].

Indomethacin, a non-corticosteroid drug, is also frequently used due to its anti-inflammatory, anti-pyretic, and pain-relieving effects [80]. However, it can cause some adverse effects, such as ulcerative lesions and erosions in the mucosa of the stomach. In indomethacin-induced gastric mucosal injury in rats, AST (25 mg/kg BW; three days) increased the activities of SOD, CAT, and GPX that provide defenses against oxidative damage in gastric mucosa [106]. According to the data in the study, AST removed the lipid peroxides and free radicals induced by indomethacin and prevented the indomethacin-induced gastric hemorrhagic lesions.

Ethanol rapidly passes through the gastric mucosa, leading to membrane injury and endothelial damage in vessels, and a subsequent increase in mucosal permeability [107]. The vascular and microvascular changes are an early event in the development of gastric ulcer [108]. In rats, AST pretreatment (5 or 25 mg/kg BW; three days) significantly increased the activity of anti-oxidant enzymes such as SOD, CAT, and GPX in response to the ethanol-induced gastric ulcer [109]. Furthermore, AST may modulate gastric acid and mucin secretion. In rats administered with ethanol, AST pretreatment (100, 250, or 500 µg/kg BW; three weeks) blocked the gastric H^+^, K^+^-ATPase proton pump, and upregulated mucin content, which protected the gastric mucosal layer against oxidative stress and excessive secretion of gastric acid in the gastric cells [110]. Similar findings were observed in another study. In young adult ddY mice, Murata et al. reported that pretreatment with AST (30 or 100 mg/kg BW; 1 h) before administering ethanol/hydrochloride reduced the total lesion area and inhibited disruption of the superficial regions of the gastric gland with epithelial cell loss. Pretreatment with AST decreased levels of lipid peroxidation and histological damage in the superficial layers of the gastric mucosa [111].

**Figure 1:**
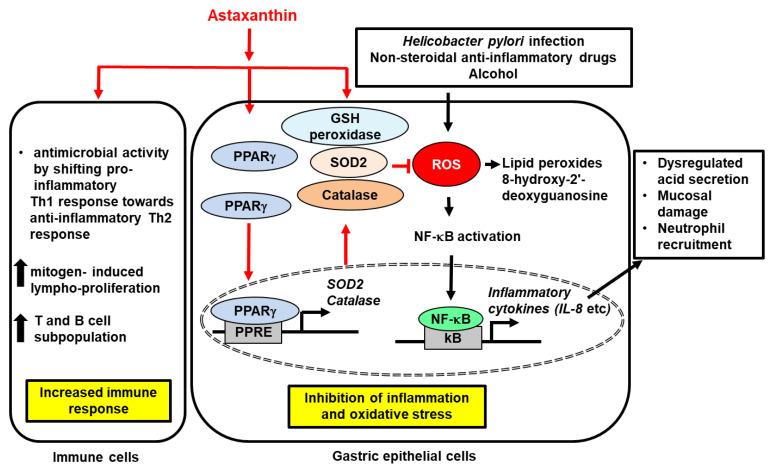
The proposed mechanism of astaxanthin-mediated protective effects on gastric ulcer. *H. pylori* infection, chronic alcohol consumption, and ingestion of nonsteroidal anti-inflammatory drugs can cause gastric ulcer development. Astaxanthin exerts anti-ulcer activity by its anti-oxidant and anti-inflammatory effects. For antioxidant activity, astaxanthin enhances the activity of anti-oxidant enzymes such as glutathione (GSH) peroxidase, superoxide dismutase (SOD), and catalase. These enzymes reduce the levels of reactive oxygens species (ROS) which increase the levels of lipid peroxides and 8-hydroxy-2′-deoxyguanosin. ROS activate nuclear factor-κB (NF-κB) to induce expression of inflammatory cytokines including interleukin (IL)-8. These cytokines result in dysregulated acid secretion, mucosal damage, and neutrophil recruitment in gastric mucosal tissues. Astaxanthin inhibits these ROS-mediated alterations and mucosal damage. In addition, astaxanthin activates peroxisome proliferator-activated receptor-γ (PPAR-γ) to induce expression of SOD2 and catalase that reduces ROS in gastric epithelial cells. In immune cells, astaxanthin shows antimicrobial activity by shifting pro-inflammatory T helper type 1 (Th1) response towards anti-inflammatory Th2 response. It stimulates mitogen-induced lympho-proliferation and increases T and B cell subpopulation. Thus, astaxanthin shows inhibitory effect on gastric ulcer development. Inhibit, 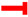; increase, 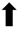. Red arrows represent the effects of astaxanthin.

The effects of AST have also been examined in a stress-induced gastric ulcer model, where stress was induced by immersing rats in chest-level water for 24 h [112]. In response to water-induced stress, rats pre-administered with AST (40 mg/100 g diet; six days) showed markedly attenuated ulcer indexes and stress-related gastric injuries compared to control-treated rats. Collectively, these results suggest that AST could be utilized as a remedy for gastric mucosal damage and ulcers. Figure 1 shows the proposed mechanism of AST-mediated protective effects on gastric ulcers.

## 3. Gastric Cancer

Despite its occurrence having declined over the past decade, gastric cancer is still one of the leading causes of cancer-related death worldwide [113]. Although the actual cause of gastric cancer is unclear, there are several risk factors associated with the disease including *H. pylori* infection, cigarette smoking, and dietary factors such as highly salted foods. *H. pylori* is classified as a group 1 carcinogen according to the World Health Organization (WHO, Geneva, Switzerland) [114]. Smoking is another risk factor for gastric cancer, as nicotine is strongly associated with gastric cancer metastasis by increasing IL-8 expression via the activation of ROS/NF-κB and ROS/MAPK (ERK 1/2, p38)/AP-1 axes in AGS gastric cancer cells, which stimulates angiogenesis and endothelial cell proliferation [115]. Furthermore, nicotine induces epithelial-mesenchymal transition (EMT) and stimulates the cyclooxygenase-2 (COX-2)/prostaglandin E2 (PGE2) signaling pathway and expression of endothelial nitric oxide synthase (eNOS) in different cancer cells [116,117,118]. A meta-analysis study reported that alcohol consumption can also increase the risk of gastric cancer [119]. It is well-known that oxidative stress and concomitant inflammation are highly involved in the tumorigenesis of gastric cancer by inducing damage to the cell membrane, DNA, and protein [120]. ROS regulate p38 MAPK, leading to cell apoptosis and the activation of various downstream signaling targets such as Wnt, Ras, mTOR, and p53 to initiate gastric carcinogenesis [121,122]. In addition, increased ROS activate redox-sensitive transcription factors such as NF-κB and AP-1, leading to the enhanced expression of inflammatory genes and adhesion molecules to promote gastric cancer cell invasion [123,124].

### The Effect of AST on Gastric Cancer

AST has been shown to suppress the migration, invasion, and proliferation of various cancer cell lines including breast, colon, lung, and gastric cancers [125,126,127]. In human gastric adenocarcinoma cell lines KATO-III and SNU-1, AST treatment (1, 10, 50, or 100 µM; 48 h) inhibited cellular proliferation by interrupting cell cycle progression [125]. This anti-proliferative effect by AST was mechanistically mediated by the inhibition of the phosphorylation of ERK and the elevated expression of p27. These events reduced the levels of the cell cycle regulators such as cyclin D1/cyclin-dependent kinase 4 (CDK4) and cyclin E/CDK2 complexes, which induced cell cycle arrest at the G0/G1 phase [125].

The anti-proliferative effect of AST is also observed in the *H. pylori*–induced gastric cancer model. RNA-sequencing (RNA-Seq) analysis for *H. pylori*-infected human gastric epithelial AGS cells revealed that AST treatment (5 µM; 3 h) significantly suppressed the *H. pylori*-induced overexpression of several genes (Fos-like 1, c-myc, and porcupine) related to the Wnt/β-catenin signaling pathway, which regulates cell proliferation [128]. At the same time, AST effectively reversed the *H. pylori*-induced downregulation of some genes including Bambi and Smad4, which function as tumor suppressors in gastric epithelial cells. In addition, AST repressed *H. pylori*-mediated Smox expression, thereby inhibiting oxidative DNA damage in gastric epithelial cells [128]. Further transcriptional array profiling in *H. pylori*-infected AGS cells identified that AST administration (5 µM; 3 h) significantly reduced expressions of c-MET, EGFR, PI3KC2, PLCγ1, Cdc42, and ROCK1, which are genes that modulate cytoskeleton reorganization, motility, and/or migration [129].

**Figure 2:**
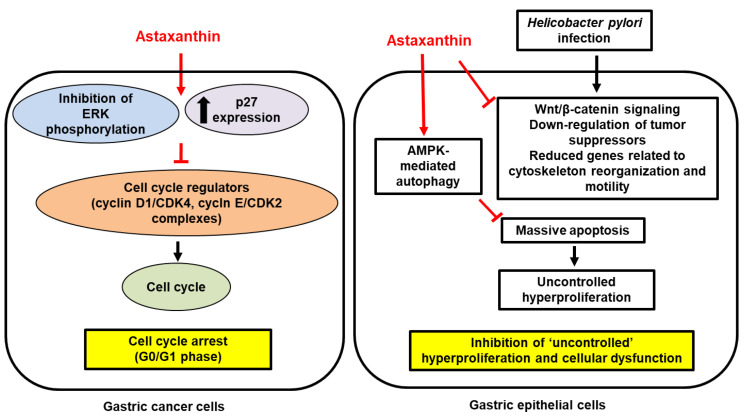
The inhibitory effect of astaxanthin on hyperproliferation and cellular dysfunction in gastric cancer development and progression. In gastric cancer cells, astaxanthin inhibits phosphorylation of extracellular signal-regulated kinase (ERK) but stimulates expression of tumor suppressor p27 that inhibits the formation of cell cycle regulators such as cyclin D1/cyclin-dependent kinase 4 (CDK4) and cyclin E/CDK2 complexes, leading to cell cycle arrest at G0/G1 phase. In *Helicobacter pylori*-infected gastric epithelial cells, astaxanthin increased 5’ adenosine monophosphate-activated protein kinase (AMPK)-mediated autophagy, resulting in inhibition of massive apoptosis and uncontrolled hyperproliferation. *Helicobacter pylori* infection activates Wnt/β-catenin signaling pathway. However, it decreases expression of tumor suppressors and genes related to cytoskeleton reorganization and motility. Therefore, astaxanthin inhibits hyperproliferation and cellular dysfunction in the pathogenesis of gastric cancer development. Inhibit, 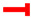; increase, 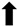. Red arrows represent the effects of astaxanthin.

In addition, AST has been shown to protect against gastric cancer via the regulation of apoptosis and autophagy. Increases in the rate of apoptotic cell death by *H. pylori* infection in gastric epithelial cells [130] may contribute to adenocarcinoma since massive apoptosis can lead to an uncontrolled hyperproliferation as a cellular homeostatic mechanism [131]. Therefore, it is critical to maintain normal apoptotic signaling to avoid uncontrolled tissue growth and neoplasia. It has been suggested that autophagy, an intracellular recycling process that modulates various cellular physiological process, can potently suppress *H. pylori*-induced apoptotic cell death by activating AMP-activated protein kinase (AMPK) signaling [132,133]. In AGS cells, Lee et al. found that AST pretreatment (25 or 50 nM; 3 h) prevented *H. pylori*-induced apoptosis through AMPK-mediated autophagy [134]. As a result, AST treatment markedly inhibited *H-pylori*-induced cell death, DNA fragmentation, the activity of caspase-3, and the release of cytochrome c in the cells. Mechanistically, AST activated AMPK activation, which, in turn, suppressed mTOR, triggering a signal to initiate autophagosome formation [134]. No clinical trials have been conducted. Figure 2 shows the proposed mechanism of AST-mediated protective effects on gastric cancer development and progression.

## 4. Ulcerative Colitis

Ulcerative colitis is a chronic inflammatory bowel disorder of which incidence is increasing worldwide [135]. Ulcerative colitis is characterized by continuous mucosal inflammation ranging from the rectum to the proximal colon, and affected patients often present diarrhea and blood in their stool [136]. The pathogenesis of ulcerative colitis is multifactorial, which includes genetic predisposition, abnormal immune responses, defects in the gut barrier, and environmental factors such as diet [137]. Excess oxidative stress produced by neutrophil accumulation, the activation of pro-inflammatory transcription factors and cytokines, and increased intestinal permeability in epithelial crypts and intestinal mucosa are the major hallmarks of ulcerative colitis [138,139].

### The Effects of AST on Ulcerative Colitis

In cyclophosphamide-induced immunodeficient mice, AST administration by oral gavage (30–120 mg/kg BW; 30 days) significantly increased the SOD, GSH, and GPX levels and decreased the MDA level, indicating that AST treatment was efficient in relieving oxidative stress [140]. The AST-mediated event coincided with stimulated goblet cell growth, mucous and IgA secretion, and reduction in antimicrobial peptides, which led to significantly ameliorated intestinal mucosa damage in immunodeficient mice [140]. In addition, supplementation of AST-enriched yeast (120 mg/kg BW; 7–21 days) enhanced the levels of immunoglobulin A (IgA) in the jejunum and ileum of weanling mice [141]. In the intestinal microflora, IgA antibodies function to defend against commensal intestinal bacteria [142]. Therefore, these findings in an immunodeficient mouse model and weanling mouse model indicate that AST has the capacity to assist with the immune system of the intestine, which is one of the largest immune organs [141,143].

In a DSS-induced colitis mouse model, the dietary pretreatment of AST (0.02–0.04% mixed in a rodent chow diet; seven days) prior to the DSS administration significantly mitigated DSS-induced body weight loss and suppressed the mucosal gene expression of pro-inflammatory cytokines including IL-1β, IL-6, TNF-α, and IL-36 [144]. Mechanistically, AST inhibited the DSS-mediated translocation of NF-κB p65 and c-Jun into the nucleus of epithelial cells in mucosa, and blocked mucosal activation of ERK1/2, p38, and JNK. Yasui et al. also reported similar results. In DSS-induced colitis, mice that were fed experimental diets containing AST (100–200 ppm) exhibited lowered gene expressions of several inflammatory markers including TNF-α, IL-1β, IL-6, COX-2, and iNOS and inhibited NF-κB expression in the colon [43]. As the applications of AST are limited by its poor water-solubility and loss of bioactivity during the digestion process, some studies utilized an encapsulation system to improve the bioavailability of AST. Zhang et al. prepared an AST-enriched colon-targeted alginate particle (AST-Alg) and tested its efficacy in mice with DSS-induced ulcerative colitis [145]. Compared to the DSS-treated control group, mice that received AST-Alg (30 ppm of AST; nine weeks) showed lower levels of inflammation, oxidative damage, colonic mucosal integrity, and body weight loss. It is noteworthy that AST-Alg displayed a better protective effect on the colitis treatment compared to an oil-in-water emulsion form of AST, suggesting that a tissue-targeted delivery system could augment the potency of AST. In another study conducted by Zhang et al., AST-loaded nanocarriers utilizing cauliflower-like carriers were prepared, and the particles were examined in in vitro and DSS-treated mice [146]. In vitro studies showed that AST-loaded nanocarriers more effectively targeted mitochondria than a free form of AST, which significantly improved the internalization of AST and reduced ROS production in the mitochondria. In vivo studies using DSS-treated BALB/c mice further showed that AST-loaded nanocarriers (250 mg/kg; 13 days) protected the colon tissue integrity by inhibiting expressions of IL-1β, IL-6, COX-2, MPO, and iNOS and by activating the expression of zonula occludens-1 (ZO-1), which is an intestinal tight junction protein [146].

**Figure 3:**
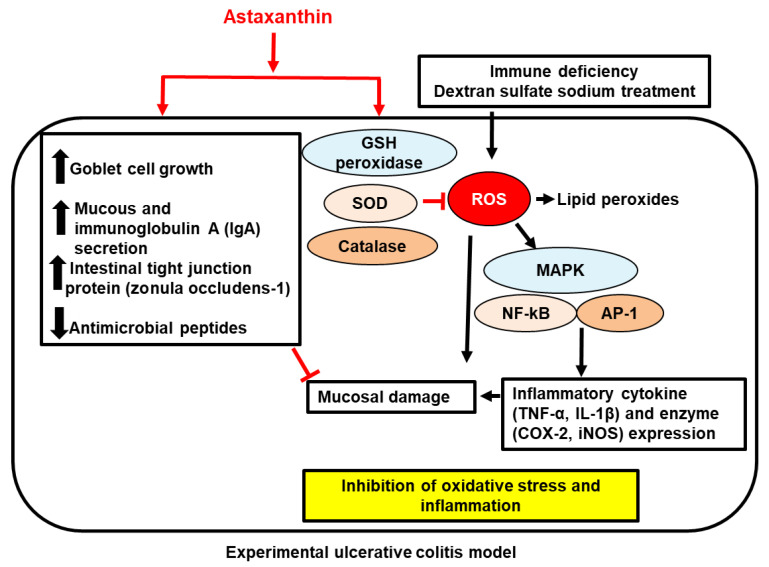
The proposed mechanism of astaxanthin-mediated protective effects on ulcerative colitis. In experimental ulcerative colitis model, induced by treatment of dextran sulfate sodium or immune deficiency, astaxanthin increased glutathione (GSH) peroxidase, superoxide dismutase (SOD), and catalase which decreases reactive oxygens species (ROS) and lipid peroxide production. Therefore, astaxanthin inhibits ROS-mediated activation of mitogen-activated protein kinases (MAPK), nuclear factor-κB (NF-κB), and activator protein 1 (AP-1) to induce expression of inflammatory cytokines such as tumor necrosis factor-α (TNF-α) and interleukin (IL)-1β, and inflammatory enzymes including cyclooxygenase-2 (COX-2) and inducible nitric oxide synthase (iNOS), which stimulates mucosal damage. Thus, astaxanthin inhibits oxidative stress-mediated inflammation. Moreover, astaxanthin stimulates goblet cells growth and secretion of mucous and immunoglobulin A (IgA), and expression of zonula occludens-1 which is intestinal tight junction protein; however, it reduces anti-microbial peptides. Overall, astaxanthin ameliorates intestinal mucosal damage. Inhibit, 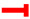; increase, 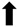; decrease, 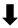. Red arrows represent the effects of astaxanthin.

AST may also be effective in treating necrotizing enterocolitis, which is a disease that is mainly observed in preterm newborns [147]. In an experimental necrotizing enterocolitis rat model, Akduman et al. reported that AST (100 mg/kg BW; four days; oral gavage) potently reduced intestinal damage due to reduced oxidative stress (higher levels of GSH and SOD), inflammation (lower levels of IL-1β and TNF-α), and apoptosis (lower levels of caspase-3) [143]. As a result, AST-administered rats showed a better survival rate and weight gain in response to necrotizing enterocolitis. Overall, these findings in rodent models suggest that AST suppresses ulcerative colitis through the inhibition of inflammation and oxidative stress, raising the possibility of nutraceutical potential in this disease. However, no clinical trials have been conducted. Figure 3 demonstrates the proposed mechanism of AST-mediated protective effects on ulcerative colitis.

## 5. Colon Cancer

Colon cancer has become the third most common cancer in men and women [114]. Although colon cancer can be inherited, several epidemiological studies have shown that the risk of colon cancer is largely determined by environmental factors such as high alcohol consumption, low-fiber diet, high-fat diet, and smoking tobacco [148,149]. In addition, studies have well-established the molecular links between chronic inflammation and oxidative stress, and colorectal carcinogenesis [150,151]. Although the molecular mechanisms by which ROS and inflammation promote carcinogenesis are still being elucidated, redox-sensitive transcription factors such as NF-κB, AP-1, and STAT3 have been strongly implicated in colon carcinogenesis [152]. Therefore, various chemoprotective agents, anti-oxidants, and anti-inflammatory bioactive compounds have been identified to provide molecular strategies for colon cancer by modulating abnormally activated NF-κB, AP-1, and STAT3.

### The Effects of AST on Colon Cancer

AST has been shown to exhibit anti-cancer effects in various types of cancers such as gastric cancer, breast cancers and prostate cancer [94,153,154]. In multiple models of colon cancer, AST has been shown to regulate hallmarks of cancer including proliferation, metastasis, and apoptosis [155]. In WiDr colon cancer cells, the administration of AST (1.25–250 µM; 72 h) exerted the cytoprotective effects by inhibiting cell proliferation [156]. Similarly, treatment of AST-rich *Haematococcus pluvialis* extract (25 µL/mL; 24 h) in HCT-116 colon cancer cells significantly increased p53, p21, and p27 expression and suppressed cyclin D1 expression and AKT phosphorylation [157]. In addition, *Haematococcus pluvialis* extract increased the phosphorylation of p38, JNK, and ERK1/2, while it modified the ratio of Bax/Bcl-2. These results indicate that AST may effectively inhibit colon cell growth by inhibiting cell cycle progression and enhancing apoptosis [157].

AST treatment appears to be highly effective in colitis-associated cancerization, which is a relatively minor risk factor. In an azoxymethane (AOM)/DSS-mediated colon carcinogenesis mouse model, the dietary administration of AST (200 ppm; 17 weeks) ameliorated colonic proliferative lesions, possibly via the inhibition of NF-κB, TNF-α, and IL-1β expressions in the malignancies [43]. Tanaka et al. also reported similar results, where an AST-containing diet (500 ppm; 34 weeks) markedly inhibited the development of aberrant crypt foci via inhibition of cell proliferation activity [158]. In rats treated with 1,2 dimethylhydrazine (DMH) to induce colon carcinogenesis, a pre-treatment of AST (15 mg/kg BW; 16 weeks) markedly decreased the severity of lesions as well as the number of aberrant crypt foci [159]. Mechanistically, AST-treated rats showed increased levels of anti-oxidants such as SOD, CAT, and GPX as well as decreased levels of lipid peroxidation. In the same experimental model, AST-administered rats (15 mg/kg BW; 16 weeks) exhibited significantly inhibited the expression of NF-κB-p65, COX-2, matrix metalloproteinase (MMP) 2/9, proliferating cell nuclear antigen (PCNA) and ERK2 induced by DMH [160]. Consistent with these observations, AST also ameliorated obesity-associated colorectal carcinogenesis. In leptin receptor-null C57BL/KsJ-db/db mice injected with AOM, which causes spontaneous obesity, Kochi et al. demonstrated that a diet containing AST (200 ppm; eight weeks) reduces oxidative stress by stimulating the expression of SOD1, GPX, and CAT in the colonic mucosa [161]. AST-fed db/db mice also showed reduced numbers of NF-κB^+^ and PCNA^+^ cells and decreased gene expressions of IL-6, IL-1β, F4/80, chemokine (C-C motif) ligand 2 (CCL2), and chemokine (C-X-C motif) ligand 2 (CXCL2) in the colon.

**Figure 4:**
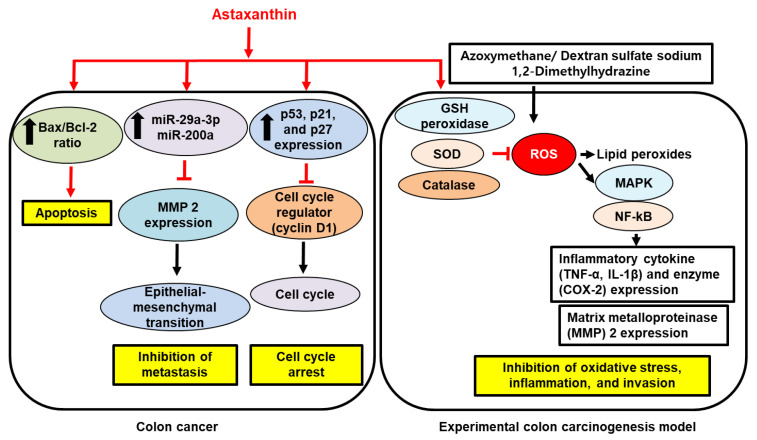
The inhibitory effect of astaxanthin on proliferation, metastasis, and oxidative stress-mediated inflammation and invasion in colon cancer development and progression. In colon cancer cells, astaxanthin increases Bax/Bcl-2 ratio, resulting in apoptosis. Astaxanthin increases micro RNA (miR)-29a-3p and miR-200a which inhibits matrix metalloproteinase (MMP) 2 expression that mediates epithelial-mesenchymal transition. Astaxanthin stimulates expression of tumor suppressor p53, p21, and p27 that inhibits the expression of cell cycle regulator cyclin D1, leading to cell cycle arrest. In experimental colon carcinogenesis model, induced by treatment of azoxymethane/dextran sulfate sodium or 1,2 dimethylhydrazine, astaxanthin increased glutathione (GSH) peroxidase, superoxide dismutase (SOD), and catalase which reduces reactive oxygens species (ROS) and production of lipid peroxides. Thus, astaxanthin suppresses ROS-mediated activation of mitogen-activated protein kinases (MAPK) and nuclear factor-κB (NF-κB) and inhibits expression of inflammatory cytokines tumor necrosis factor-α (TNF-α) and interleukin (IL)-1β, inflammatory enzyme cyclooxygenase-2 (COX-2), and MMP2. Therefore, astaxanthin inhibits inflammation and invasion by reducing ROS levels in the pathogenesis of colon cancer development. Inhibit, 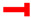; increase, 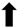. Red arrows represent the effects of astaxanthin.

Anti-cancer activity of AST could also be mediated by modulating microRNAs (miR). In colon cancer cell lines CT26 and HCT-116, AST treatment (50–100 µM; 24 h) transcriptionally repressed an oncogenic transcriptional factor, MYC, and thereby enhanced the expression of miR-29a-3p and miR-200a, which are known to exhibit an anti-metastatic effect [162]. In turn, the increased miR-29a-3p and miR-200a resulted in the reduced expression of MMP2 and zinc finger E-box binding homeobox 1 (ZEB1), which mediate the epithelial-mesenchymal transition of colorectal cancer cells. These mechanisms were also confirmed in colon cancer cell-injected BALB/c nude mice, where AST administration (25–50 mg/kg BW; four weeks) inhibits the metastasis of colon cancer to the lung via the MYC/miR-29a-3p and miR-200a axis [162]. Overall, these findings suggest that AST has anti-oxidant, anti-inflammatory, and anti-metastasis effects against colon cancer progression. AST could be a potential candidate for a chemoprevention agent of the disease. However, no human clinical trials have been tried. Figure 4 shows the proposed mechanism for the inhibitory effect of AST on proliferation, metastasis, and oxidative stress-mediated inflammation and invasion in colon cancer development and progression.

## 6. Conclusions

In this review, the anti-oxidative and anti-inflammatory properties of AST have been highlighted in various experimental models of GI ulcers and cancers. Locating at the surface and on the inside of cellular membranes, AST functions as an anti-oxidant by robustly neutralizing ROS and lipid peroxyl radicals [49,51]. Moreover, AST also enhances the activity of anti-oxidant enzymes such as SOD, CAT, and GPX [105,106,109]. As an inflammatory modulator, AST regulates inflammatory signaling pathways such as NF-κB, AP-1, and MAPK and concomitantly downregulates the expression of pro-inflammatory cytokines such as IL-6, IL-1β, IL-8, and TNF-α [43,92,144]. In GI cancer models, AST appears to inhibit cancer cell growth and metastasis by modulating cell proliferation-related signaling pathways as well as apoptosis, autophagy, and miRNAs [125,134,162]. This was further evidenced by a RNA-Seq analysis in gastric cancer cells, where the profiling revealed several target genes of AST which are associated with cancer cell proliferation and metastasis [128,129]. Considering its safe profile as a bioactive compound [163], AST may become an effective and non-toxic treatment for these GI diseases. Well-organized clinical trials should be performed to determine the effect of AST against GI diseases in humans.

## Data Availability

The data used to support the findings of this study are included within the article.
